# LATER LIFE AS A DARING EXPERIENCE: FACTORS ASSOCIATED WITH OLDER ADULTS’ RISK PERCEPTION

**DOI:** 10.1093/geroni/igad104.3035

**Published:** 2023-12-21

**Authors:** Rinat Lifshitz, Yaacov Bachner, Galit Nimrod

**Affiliations:** The Max Stern Academic College of Emek Yezreel, Israel, Yezreel Valley, HaZafon, Israel; Ben-Gurion University of the Negev, Beer-Sheva, HaDarom, Israel; Ben-Gurion University of the Negev, Beer-Sheva, HaDarom, Israel

## Abstract

Risk perception refers to people’s subjective judgments about the possibility of negative occurrences and the extent to which they are concerned with them. Previous studies have found that older adults who were exposed to ongoing terror threats developed later-life and terror risk perceptions. These studies showed that high risk perception has negative psychological and physiological consequences. This study aimed to identify the factors associated with the development of both later-life risk perception and terror risk perception in later life. Data were collected via an online survey with 306 Internet users aged 50 years and over, half resided in a high-risk zone while the remainder lived in a low-risk zone. The Perceived Risk Scale, measures of depressive symptoms, life satisfaction, social support, spirituality, internet use, and personal background were applied. Low self-rated health was associated with terror and later-life risk perceptions, regardless of the risk zone. After controlling for personal background, only depressive symptoms significantly correlated with high risk perceptions. These findings suggest that older adults with poorer self-rated health, secular beliefs, and elevated depressive symptoms may be susceptible to developing high risk perceptions. Clinicians should encourage older adults to identify preserving resources to improve adjustment to late life stressors.

